# Interim Estimates of 2022–23 Seasonal Influenza Vaccine Effectiveness — Wisconsin, October 2022–February 2023

**DOI:** 10.15585/mmwr.mm7208a1

**Published:** 2023-02-24

**Authors:** Huong Q. McLean, Joshua G. Petrie, Kayla E. Hanson, Jennifer K. Meece, Melissa A. Rolfes, Gregg C. Sylvester, Gabriele Neumann, Yoshihiro Kawaoka, Edward A. Belongia

**Affiliations:** ^1^Marshfield Clinic Research Institute, Marshfield, Wisconsin; ^2^Influenza Division, National Center for Immunization and Respiratory Diseases, CDC; ^3^CSL Seqirus, Summit, New Jersey; ^4^Department of Pathobiological Sciences, School of Veterinary Medicine, University of Wisconsin-Madison, Madison, Wisconsin.

In the United States, 2022–23 influenza activity began earlier than usual, increasing in October 2022, and has been associated with high rates of hospitalizations among children* (*1*). Influenza A(H3N2) represented most influenza viruses detected and subtyped during this period, but A(H1N1)pdm09 viruses cocirculated as well. Most viruses characterized were in the same genetic subclade as and antigenically similar to the viruses included in the 2022–23 Northern Hemisphere influenza vaccine (*1*,*2*). Effectiveness of influenza vaccine varies by season, influenza virus subtype, and antigenic match with circulating viruses. This interim report used data from two concurrent studies conducted at Marshfield Clinic Health System (MCHS) in Wisconsin during October 23, 2022–February 10, 2023, to estimate influenza vaccine effectiveness (VE). Overall, VE was 54% against medically attended outpatient acute respiratory illness (ARI) associated with laboratory-confirmed influenza A among patients aged 6 months–64 years. In a community cohort of children and adolescents aged <18 years, VE was 71% against symptomatic laboratory-confirmed influenza A virus infection. These interim analyses indicate that influenza vaccination substantially reduced the risk for medically attended influenza among persons aged <65 years and for symptomatic influenza in children and adolescents. Annual influenza vaccination is the best strategy for preventing influenza and its complications. CDC recommends that health care providers continue to administer annual influenza vaccine to persons aged ≥6 months as long as influenza viruses are circulating (*2*).

VE against medically attended influenza was estimated using a test-negative case-control design. Patients aged 6 months–64 years were actively recruited during or after outpatient medical care for ARI (i.e., telehealth, primary care, urgent care, or emergency department), and before or during appointments for clinical testing for SARS-CoV-2 at selected MCHS facilities. Patients were eligible if they had a cough of ≤7 days’ duration and had not taken an influenza antiviral medication. Participants completed a brief survey and provided a respiratory specimen for influenza and SARS-CoV-2 testing. Participants who received a positive real-time reverse transcription–polymerase chain reaction (RT-PCR) test result for SARS-CoV-2 were excluded from VE estimation. Participants were considered vaccinated if MCHS health records indicated receipt of seasonal influenza vaccine according to Advisory Committee on Immunization Practices (ACIP) recommendations ≥14 days before illness onset^†^ (*2*). VE against influenza A viruses and against influenza A(H3N2) viruses was estimated as 100% x (1 − adjusted odds ratio [aOR]). The aOR is the ratio of the odds of vaccination among those who received a positive influenza test result (case-patients) to the odds of vaccination among those who received negative test results for influenza and SARS-CoV-2 (control-patients). Estimates were adjusted for age, month of illness onset, and self-reported presence of one or more higher-risk condition^§^ using logistic regression.

VE against symptomatic influenza in children and adolescents was estimated from an ongoing, prospective, community-cohort study in central Wisconsin (*3*). Each week, participants (or their guardians) reported the absence or presence of specific symptoms during the previous 7 days. An anterior nasal swab was self- or guardian-collected for research testing when participants reported one or more of the following: fever, cough, loss of smell or taste, sore throat, muscle or body aches, shortness of breath, diarrhea, nasal congestion or runny nose, or nausea or vomiting. Influenza infection was defined as a positive result from research testing or a positive result from clinical testing (results extracted from MCHS health records). Unvaccinated person-time was defined as the time from October 23, 2022 (7 days before occurrence of the first influenza case), until receipt of seasonal influenza vaccine. Vaccinated person-time began ≥14 days after receipt of influenza vaccine (based on health records) according to ACIP recommendations. Person-time for the 13 days after receipt of vaccine was excluded from the analysis. Hazard ratios comparing the rate of influenza A virus infection among vaccinated and unvaccinated participants were estimated using Cox proportional hazards models with time-varying influenza vaccination status, age, the presence of one or more higher-risk condition, and COVID-19 vaccination status. VE was estimated as 100% x (1 − adjusted hazard ratio). Influenza and SARS-CoV-2 RT-PCR testing and genetic characterization of influenza-positive specimens for both studies were performed at MCHS research laboratory. Protocols for both studies were reviewed and approved by the Institutional Review Board at MCHS and were conducted consistent with applicable federal law and CDC policy.^¶^

During December 2, 2022–February 10, 2023, a total of 545 children, adolescents, and adults with medically attended ARI were included in the test-negative design case-control study; 116 (21%) received a positive test result for influenza A virus, and none received a positive test result for influenza B virus. Among 115 (99%) influenza A virus subtypes determined, 29 (25%) were A(H1N1)pdm09 viruses, and 86 (75%) were A(H3N2) viruses (Table 1). All of the 43 characterized viruses were genetically similar to vaccine components; 34 A(H3N2) viruses belonged to subclade 2a.2 and nine A(H1N1)pdm09 viruses belonged to subclade 5a.2. The proportion of patients with influenza differed by month of illness onset. Among ARI patients, 186 (34%) had documentation of receipt of 2022–23 influenza vaccine; the percentage vaccinated differed by sex, higher-risk condition, and COVID-19 vaccination status. A large majority of vaccinated participants (84%) received cell culture–based vaccine (ccIIV4). Among the 116 participants who received a positive influenza test result, 26 (22%) received the 2022–23 seasonal influenza vaccine, compared with 160 (37%) of 429 participants who received negative test results for influenza and SARS-CoV-2 (Table 2). The overall adjusted VE against outpatient medically attended ARI associated with influenza A was 54% and 60% against influenza A(H3N2) viruses.

**TABLE 1:** Selected characteristics of enrolled patients with medically attended acute respiratory illness and participants of a community cohort, by influenza test result and seasonal influenza vaccination status — Wisconsin, October 2022–February 2023

Characteristic	Test-negative case-control study*				Community cohort study^†^		
	No. of participants	No. (%)			No. of participants	No. (%)	
		Vaccinated^§^	Positive influenza test result	Negative influenza and SARS-CoV-2 test results		Vaccinated^¶^	Positive influenza test result
**Total**	**545**	**186 (34)**	**116 (21)**	**429 (79)**	**241**	**94 (39)**	**34 (14)**
Age group**							
6 mos–17 yrs	223	69 (31)	42 (19)	181 (81)	241	94 (39)	34 (14)
18–64 yrs	322	117 (36)	74 (23)	248 (77)	NA	NA	NA
**Sex**							
Female	318	127 (40)	65 (20)	253 (80)	116	49 (42)	17 (15)
Male	227	59 (26)	51 (22)	176 (78)	125	45 (36)	17 (14)
**Race and ethnicity**							
Hispanic or Latino	35	12 (34)	6 (17)	29 (83)	2	2 (100)	1 (50)
White, non-Hispanic	482	161 (33)	105 (22)	377 (78)	233	92 (39)	33 (14)
Other races, non-Hispanic^††^	28	13 (46)	5 (18)	23 (82)	6	0 (—)	0 (—)
**Higher-risk conditions** ^§§^							
Yes	154	69 (45)	30 (19)	124 (81)	31	13 (42)	6 (19)
No	391	117 (30)	86 (22)	305 (78)	210	84 (39)	28 (13)
**≥2 COVID-19 vaccine doses^¶¶^**							
Yes	258	133 (52)	51 (20)	207 (80)	115	61 (53)	20 (17)
No	287	53 (18)	65 (23)	222 (77)	126	33 (26)	14 (11)
**Month of illness onset**							
Nov–Dec 2022	227	75 (33)	86 (38)	141 (62)	NA	NA	32 (94)
Jan–Feb 2023	318	111 (35)	30 (9)	288 (91)	NA	NA	2 (6)
**Influenza test result**							
Negative	429	160 (37)	NA	429 (100)	207***	88 (43)	NA
Influenza A–positive	116	26 (22)	116 (100)^†††^	NA	34	6 (18)	34 (100)^†††^
A(H3N2)	86	16 (19)	86 (74)^†††^	NA	29	5 (17)	29 (85)^†††^
A(H1N1)pdm09	29	10 (34)	29 (25)^†††^	NA	1	0 (—)	1 (3)^†††^
A: unknown subtype	1	0 (—)	1 (1)^†††^	NA	4	1 (25)	4 (12)^†††^

**Abbreviations**: ACIP = Advisory Committee on Immunization Practices; ccIIV4 = cell culture–based vaccine; MCHS = Marshfield Clinic Health System; NA = not applicable.

* A total of 109 participants received a positive test result for SARS-CoV-2 virus infection and were excluded. Participants with uncertain influenza vaccination status (12), with receipt of vaccine ≤13 days before illness (four), or who were aged <9 years and partially vaccinated (seven) were excluded from analysis.

^†^ One child was partially vaccinated according to ACIP recommendations before the analysis period and was excluded.

^§^ Defined as receipt of any seasonal influenza vaccine according to ACIP recommendations ≥14 days before illness onset based on MCHS vaccination records. Most vaccinated participants (84%) received ccIIV4.

^¶^ Defined as receipt of seasonal influenza vaccine according to ACIP recommendations ≥14 days before influenza infection or before the end of follow-up based on MCHS vaccination records. Most vaccinated participants (84%) received ccIIV4.

** Age on the date of the clinical encounter for acute respiratory illness for the test-negative case-control study and as of September 1, 2022, for the community cohort study.

^††^ Includes persons who are American Indian or Alaska Native, Asian, Black or African American, Native Hawaiian or other Pacific Islander, and multiracial.

^§§^ Based on self-report of asthma or another chronic lung disease, cancer, diabetes, heart disease including high blood pressure, immunocompromising condition, kidney disease, liver disease, obesity, or pregnancy during the 12 months preceding the test-negative case-control study enrollment and self-report of asthma, immunocompromised state, serious heart condition, or other chronic lung disease for the community cohort study.

^¶¶^ Based on self-report for the test-negative case-control study and health records for the community cohort study.

*** Includes cohort participants with acute respiratory illness who received negative influenza test results, those with no reported acute respiratory illness, and three persons with influenza infections occurring within 14 days after vaccination who were excluded from the study at the time of vaccination.

^†††^ Column percentages.

**TABLE 2:** Estimated 2022–23 influenza vaccine effectiveness* — Wisconsin, October 2022–February 2023

Influenza type	Test-negative case-control study, persons aged 6 mos–64 yrs					Community cohort study, persons aged 1–17 yrs				
	Positive influenza test result		Negative influenza and SARS-CoV-2 test results		Adjusted VE,* % (95% CI)	Vaccinated		Not vaccinated		Adjusted VE,^†^ % (95% CI)
	Total	No. of persons vaccinated (%)	Total	No. of persons vaccinated (%)		No. of person-days	No. of positive influenza test results	No. of person-days	No. of positive influenza test results	
**A**	**116**	26 (22)	**429**	160 (37)	54 (23–73)	7,292	6	15,678	28	71 (31–90)
**A(H3N2)**	**86**	16 (19)	**429**	160 (37)	60 (25–79)	NE	NE	NE	NE	NE

**Abbreviations**: aOR = adjusted odds ratio; NE = not estimated; VE = vaccine effectiveness.

* VE was estimated using the test-negative design as 100% x (1 − aOR) in which aOR represents ratio of odds of being vaccinated among influenza-positive cases to odds of being vaccinated among influenza-negative and SARS-CoV-2–negative controls; odds ratios were estimated using logistic regression with adjustment for age, month of illness onset, and presence of one or more higher-risk condition (self-report of asthma or another chronic lung disease, cancer, diabetes, heart disease including high blood pressure, immunocompromising condition, kidney disease, liver disease, obesity, or pregnancy in the 12 months preceding enrollment). https://www.cdc.gov/flu/vaccines-work/us-flu-ve-network.htm

^†^ VE was estimated from a Cox proportional hazards model with time-varying influenza vaccination status, age, presence of at least one higher-risk condition (self-report of asthma, immunocompromised state, serious heart condition, or other chronic lung disease), and receipt of ≥2 COVID-19 vaccine doses before the analysis period.

Among 241 community cohort participants aged 1–17 years, 94 (39%) had documented receipt of the 2022–23 influenza vaccine (Table 1); 84% received ccIIV4. Among community cohort participants who received the 2022–23 influenza vaccine, 65% had documentation of receipt of ≥2 COVID-19 vaccine doses. During October 23, 2022–February 10, 2023, 37 (15%) participants received a positive test result for influenza A virus infection; however, three of these occurred ≤14 days after influenza vaccination and were excluded from the study at the time of vaccination. Among the remaining 34 influenza virus infections included in the analysis, 29 were caused by A(H3N2),** one by A(H1N1)pdm09, and four by influenza A viruses with unknown subtype. Six children (18%) with influenza A had received the 2022–23 seasonal influenza vaccine. Among 15,678 unvaccinated person-days, 28 influenza A virus infections occurred (incidence = 1.79 per 1,000 person-days), and among 7,292 vaccinated person-days, six influenza A virus infections occurred (incidence = 0.82 per 1,000 person-days) (Table 2). VE against symptomatic influenza A virus infection was 71%.

## Discussion

Influenza activity for the 2022–23 winter season increased earlier than usual, with high rates of influenza-associated hospitalizations among children (*2*). The interim estimates of 2022–23 influenza VE from two concurrent studies in Wisconsin suggest that the current season’s influenza vaccines are providing substantial protection against influenza. These findings are consistent with estimates reported in the Southern Hemisphere for the 2022 season and Canada for the current season, where similar viruses predominated (*4*,*5*). However, influenza vaccination coverage in the United States this season has been lower than during pre–COVID-19 pandemic seasons, particularly among children, pregnant women, and in rural areas (*6*). Increased vaccination coverage is needed to realize the full potential of seasonal influenza vaccines.

The interim estimates reported reflect early season VE and might differ from end-of-season VE estimates with additional enrollments, or if a change in circulating viruses would occur later in the season. Through the week ending February 4, 2023, influenza activity was low nationally. However, CDC continues to monitor influenza activity through routine surveillance for any indications that activity might increase again; two waves of influenza activity have occurred during many previous seasons (*7*). Seasonal influenza vaccines protect against influenza A and B viruses, both of which might continue or begin to circulate later in the season, resulting in potentially serious complications.

The findings in this report are subject to at least four limitations. First, the studies were restricted to participants from a single geographic area (central Wisconsin). However, viruses that predominated in the study population were similar to those that predominated across the United States (*1*). Second, older adults aged ≥65 years were excluded from the test-negative study. Age-specific VE estimates against influenza virus infection caused by A(H3N2) viruses are generally lower for older adults (*8*). Third, sample sizes were small for the interim analysis, which limited the precision of VE estimates, and VE against illness associated with A(H1N1)pdm09 virus infections and age-specific estimates could not be determined. Finally, confounding and bias are of concern with observational studies; however, estimates were comparable across two study designs, and the test-negative study design yields valid estimates of influenza VE in most scenarios (*9*).

Annual influenza vaccination is the best strategy for preventing influenza and its complications. During the 2022–23 season to date, influenza A viruses that predominated are genetically and antigenically similar to current vaccine components. Interim VE estimates from this report indicate that the current season’s influenza vaccine substantially reduces the risk for medical visits among persons aged 6 months–64 years and symptomatic illness associated with influenza A virus infection among children and adolescents aged <18 years. Influenza vaccination is recommended for all persons aged ≥6 months for as long as influenza viruses are circulating in the community.

**Summary**

**What is already known about this topic?**

Effectiveness of influenza vaccine varies by season, influenza virus subtype, and antigenic match with circulating viruses.

**What is added by this report?**

Data from two concurrent studies in Wisconsin found that effectiveness of the 2022–23 influenza vaccine was 54% for preventing medically attended influenza A infection among persons aged <65 years and 71% for preventing symptomatic influenza A illness among children and adolescents aged <18 years.

**What are the implications for public health practice?**

The 2022–23 influenza vaccine provides substantial protection against circulating influenza A viruses and remains the best way to protect against influenza. Influenza vaccination is recommended as long as influenza viruses are circulating.

## Footnotes

* Routine influenza surveillance in the United States indicated that influenza viruses began to circulate and outpatient visits for influenza-like illness were increased above seasonal baseline levels in epidemiologic week 40 (the week ending October 8, 2022).

† Persons aged ≥6 months are recommended to receive annual influenza vaccination. Certain children aged 6 months–8 years need 2 doses of influenza vaccine, depending on influenza vaccination history. Persons aged ≥9 years are recommended to receive 1 dose of influenza vaccine each year, regardless of influenza vaccination history. For the test-negative case-control design analysis, children aged 6 months–8 years were excluded if they needed 2 doses and, at the time of illness, they had received only 1 dose of influenza vaccine ≥14 days earlier, meaning that they were partially vaccinated. For the cohort study, children aged 6 months–8 years were excluded from the study at the time of the first dose if they needed 2 doses and they had received only 1 dose of influenza vaccine.

§ Based on self-report of asthma or another chronic lung disease, cancer, diabetes, heart disease including high blood pressure, immunocompromising condition, kidney disease, liver disease, obesity, or pregnancy in the 12 months preceding the test-negative case-control study enrollment and self-report of asthma, immunocompromised state, serious heart condition, or other chronic lung disease for the community cohort study.

¶ 45 C.F.R. part 46; 21 C.F.R. part 56.

** Six A(H3N2) viruses from the community cohort study were genetically characterized and belonged to subclade 2a.2.
